# The Method of Computing Diameter of Nano Wood Powder Based on Geometric Figure Fitting

**DOI:** 10.3390/ma14154319

**Published:** 2021-08-02

**Authors:** Lei Zhao, Xueling Yang, Jun Ma, Jianhua Wang

**Affiliations:** 1 College of Science and Information Science, Qingdao Agricultural University, Qingdao 266109, China; 201701010@qau.edu.cn; 2 College of Computer Information Engineering, Guangzhou Huali Science and Technology Vocational College, Guangzhou 511325, China; Yangxuelinghlkj@sina.com; 3 Principal’s Office, Guangzhou Huali Science and Technology Vocational College, Guangzhou 511325, China

**Keywords:** nanometer wood powder, diameter, shape context, the hole filling

## Abstract

Computing the diameter of nanometer wood powder is the key step of intelligently acquiring wood powder mesh during processing and production in the wood powder manufacturing industry. To obtain the micro image of nano wood powder, the method of hole filling is adopted to fill the binary image of wood powder particles. The contours of wood powder particles are extracted with the use of the edge detection operator, and the control experiment is carried out accordingly. The shape line method is adopted while fitting the geometric shape of wood powder particles, and the longest side or diameter of the figure is solved so as to obtain the diameter. In addition, based on the conversion standard, the mesh number of particles is calculated. The method presented in this study is expected to facilitate the automation of wood powder pellet processing industry, whereas the method is also found to have optimal applicability and reference significance for the measurement of other sorts of particles.

## 1. Introduction

The research on the structure and properties of nanoscale materials is a vital point during the development of modern science and technology, whereas the industrial revolution marks the milestone of breakthrough in the 21st century. In the lumber industry, the nanotechnology is also expected to usher in a new round of technological revolution. It has become a critical orientation for scholars of the wood science to conduct studies on wood powder from the perspective of nano technology, thus transforming the research on wood from macro perspective to micro perspective of cells [1]. Nanometer is a unit of length abbreviated as Nm. If the nanometer with wood cells is compared in wood science, then the diameter of a wood cell is approximately 3 × 10^−6^ m, which is equivalent to roughly 30 µm and 30,000 nm [2]. In this study, the envelope diameter of nano and subnano particles of wood powder should have fallen within the range of 1000–40,000 nm. Specifically, particles with a diameter ranging between 1000 and 8000 nm are referred to as nano wood powder particles; particles with a diameter ranging between 8000 and 20,000 nm are referred to as subnano wood powder particles; particles with a diameter ranging between 20,000 and 40,000 nm are referred to as ultrafine wood powder; whereas particles with a diameter of over 40,000 nm are referred to as ordinary wood powder [3]. Varying diameters of wood powder failed to pass the application scene, such as plastic special wood powder, bamboo powder, wood powder, wood putty with high mesh powder general mesh from 50–700 mesh. In addition, the woods powder adopted in aromatherapy is generally 70–90 mesh, etc. [4].

During industrial production, it is complicated to measure the size of particles of nano wood powder. The traditional screening method features certain statistical rules, but it remains hard to detect and forecast the specific values. To meet the requirements of intelligent and automatic measurement of the size of wood powder particles during industrial production, it is necessary to identify a convenient and fast approach. Judging from the study, the method of measuring the nano particle size based on graph fitting is able to meet the needs of industrialization.

**Figure 1:**
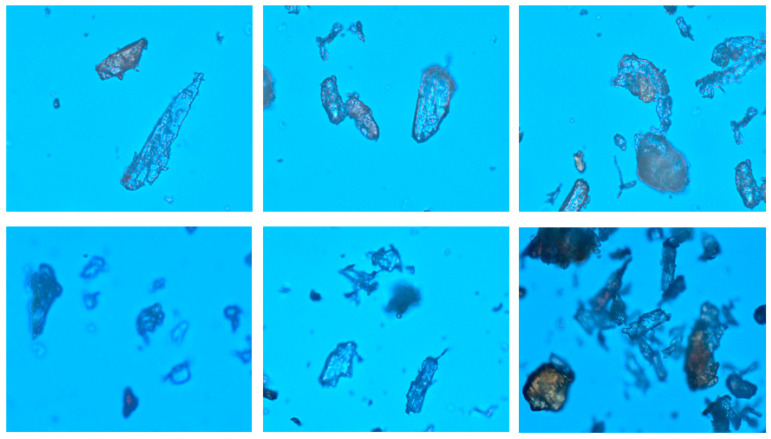
Microscopic images of wood flour.

From the macro perspective, wood powder is merely a sort of powder. However, judging from the microscopic observation, wood powder is the agglomeration of a large number of short fibers, and numerous raw materials exist during the production of wood powder. Furthermore, subsequent to the processing procedures such as crushing and shunt gas, wood powder exists in such forms as sawdust, wood shavings, scrap wood, straw and peanut shells. For instance, the fibrous structure originated from the form of wood powder particles with a rather enriched composition that mainly contains cellulose, hemicellulose and ash content and lignin. Furthermore, such wood powder is quite versatile and is regarded as a new raw material applicable to energy-saving and environmental protection [5,6,7,8]. The common microscopic image of wood powder is illustrated in Figure 1.

In the conventional procedure of preparing wood flour, the method of identifying the mesh number of wood flour typically relies on manual detection. In general, the mesh number of wood flour refers to the number of holes per square meter. The greater the mesh number of wood flour, the smaller the diameter. In addition, the diameter of wood flour could be expressed by several forms of diameter, such as screening diameter and sedimentation diameter, which is not included into the scope of this study [9,10,11,12,13].

With the progress made in the industrialization of wood flour processing, it is urgent to figure out a method capable of meeting the requirements of industrial and automatic detection of wood flour mesh number, so as to enhance the manufacturing precision and work efficiency of the technologies of processing wood flour. Judging from the examination of the microscopic image of the wood powder, the corresponding processing algorithm of digital images has been identified for handling wood powder images. Through the algorithm, the images can be obtained through calculating the wood powder diameter of wood flour mesh. In addition, the automatic monitoring of wood powder preparation becomes feasible with enhanced precision, facilitating the intelligent, automatic and high-precision production and testing of wood powder. Therefore, the study has vital implications and broad prospects of development.

## 2. Materials and Methods

### 2.1. Shape Context

Through the observation of the microscopic images of wood powder, it is found that wood powder particles are spherical or rod-shaped in general, but these particles are found to be round or rectangular when analyzed from the two-dimensional perspective of the images. Therefore, the similarity between wood powder particles of rectangular and circular shapes is analyzed first based on their shape context. Subsequently, the diameter of wood powder can be obtained in an accurate manner.

If the target shape is taken as a set of points, then it could be assumed that the shape is represented by a finite set of points in essence. In fact, the shape can be represented by a discrete set of upper sampling points interior or exterior to the object contour, which are not necessarily required to be consistent with such key points as maximum curvature and deformation points. In general, the points on the shape can be sampled by coarse equalization interval, which a rather simple method, but it should be noted that the contour-based method is not feasible for the use of the inner contour of the shape [14,15,16].

Based on the shape contour, the shape context is taken as a set of contour points $T=\left\{t_{0},t_{1},...,t_{m}\right\}$, which mainly illustrates the spatial relationship between a reference point and all the other points in the sequence of contour. For each feature point $t_{i}$ in the set of contour points, the position relationship can be calculated between other feature points in the set of contour points and $t_{i}$ through the shape context algorithm, leading to the corresponding shape histogram. Through this histogram, the information of $t_{i}$ and other feature points can be recorded correspondingly. Subsequently, the matching issue of image shapes will be transformed into the issue of identifying the corresponding point between two feature points through comparison of the histograms of two shapes.

Assuming that the contour is piecewise smooth, then an approximation of the shape can be obtained in case n is sufficiently large. Given that the shape and the results of sampling could vary from instance to instance, and that it is feasible to match points of varying classes in tandem, it is improper to regard the entire set of vectors as a shape descriptor. Therefore, the shape context descriptor is adopted in this study. The descriptor is capable of determining the distribution through the relative positions between finite sampling points, which is robust, compact and can facilitate decision-making.

For pixel $t_{i}$ in the target shape, the shape context can be obtained through depiction of the coarse histogram with other points.

(1) $$s_{j}\left(m\right)=\#\left\{t\neq s_{i}:\left(t-s\right)\in bin\left(m\right)\right\}$$

where, m∈{1,2,...,Y}. In this study, the logarithmic polar coordinate system is adopted while sampling the discrete points. Assuming that the reference direction of the coordinate system is consistent with the known axis, namely, the measurement Angle θ corresponds to the positive axis of x and that $t_{i}$ is the origin, then the polar coordinate transformation formula can be adopted, as shown in Equations (2) and (3):

(2) $$r=\sqrt{{\left(i-i_{0}\right)}^{2}+{\left(j-j_{0}\right)}^{2}}$$

(3) θ=1tan(j−j0x−x0)

Based on the shape context algorithm, the matching procedure is carried out by calculating the matching substitution value COST between the sets of two shape points. The substitution value COST between two points $t_{i}$ and $s_{j}$ in two shapes can be obtained according to the statistical results of their statistical histograms.

(4) $${\mathrm{Cos}t}_{a,b}=C\left(t_{a},s_{b}\right)=\overset{K}{\underset{k=1}{\sum }}\frac{{\left[h_{a}\left(k\right)-h_{b}\left(k\right)\right]}^{2}}{h_{a}\left(k\right)+h_{b}\left(k\right)}$$

where, $h_{a}(k)$ refers to the shape histogram of point $t_{a}$ of target T; $h_{b}(k)$ refers to the shape histogram of point $s_{b}$ of target S. Based on the formula, the cost matrix C can be obtained between the two targets with the magnitude n∗n. Through this method, the similarity of two objects can be represented by a non-vector value given that the method is based on the cost matrix. Therefore, the larger the result, the more dissimilar it is and vice versa [17,18,19]. In this study, this method has been adopted to determine the similarity between wood powder and rectangular and circular shapes.

### 2.2. Hole Filling

In graphics, processing of images and conversion of graphics into images, the region filling algorithm is deemed as a critical and fundamental algorithm [20,21]. Region filling refers to the procedure of assigning a given color to a point (usually referred to as a seed point) in an area that conforms to the definition of four-connected or eight-connected, before extending this color to the entire area.

The major deficiency of the hole filling algorithm is that it is too costly to maintain and sort all sorts of tables. As for the seed filling algorithm, the major shortcoming is that it requires a stack structure, and, accordingly, an ample storage space to facilitate the establishment of the stack structure. Furthermore, when there are multiple objects to be filled, selecting the seed points one by one risks lowering the efficiency and could be infeasible in some cases. Even though the polygonal edge mark filling algorithm is fast, it is found to be deficient while filling the arbitrary shape area. This deficiency is especially prominent in the case that the original shape is required to be maintained intact during processing of images [22].

Based on the features of wood powder particles and microscopic images, a widely applicable algorithm is adopted for the hole filling in arbitrary shape area [23,24,25]. The algorithm based on seed filling is the most common sort of algorithm for region filling. Assuming that a certain point within the closed contour is known, then other pixel points adjacent to the seed point can be identified, which are located within the contour. The overflow method is regarded as the simplest algorithm of seed filling. The procedures of processing are specified as follows: First, examining the image element of position A within the target area, so as to detect whether it is A hole (e.g., 0); if so, it is set to the region color (such as 255). The next step is to continue to iteratively detect its 4 or 8 neighborhoods, so as to realize the filling of 4 connected region or 8 connected regions. This algorithm is only applicable for filling the inner defined region. The procedures of the boundary filling algorithm are the same as those of the flood water method. The difference lies in the judgment of whether the pixel at position B is located within the region and has not been accessed. The method involves two steps: First, comparing the pixel with the boundary value of the region to check whether the pixel is a part of the region. Second, verifying whether the pixel has been accessed. The algorithm is simple in procedure, but it may lead to stack overflow due to the use of stack storage. Therefore, this method is not applicable for filling large holes. Based on the features of wood flour image, the algorithm of seed filling has been adopted in this paper.

### 2.3. Contour Extraction

Extracting the contour of wood powder particles is the prerequisite of calculating the diameter of wood powder in an accurate manner, which will further facilitate the acquisition of the mesh number of wood powder. In this study, based on the actual circumstances of high noise and low contrast of the microscopic images of wood powder particles, numerous operators have been adopted, including the Sobel operator, Roberts operator, Prewitt operator, Laplacian operator and Canny operator, which are used to process the images of nano-sized wood powder and to extract the contour of wood powder particles.

### 2.4. Minimum External Geometry

Through the calculation of the diameter of wood powder, the minimum external geometry can be obtained for wood powder particles. Specifically, the longest edge is regarded as the diameter. Therefore, the acquisition of the minimum external geometry could impose an impact on the calculation of the diameter of wood powder.

In terms of the conventional method of obtaining the minimum external geometric figure of the target, the general goal is to solve the convex contour of the target contour, and then the method of rotation or projection is adopted to acquire the minimum area figure [26,27,28]. These algorithms have their respective deficiencies judging from the study. For instance, the accuracy of the results of calculation is largely affected by the rotation angle, and to enhance the accuracy of the results, a small rotation angle will be the only option, which will also lead to a waste of system resources. The geometric envelope solution based on the hybrid genetic algorithm and simulated annealing helps solve the problem of selecting the Angle of each rotation in the normal rotation method, but this method is extremely time-consuming and it is hard to meet the real-time demand. During the feature extraction, it is oftentimes expected to obtain other quantities related to geometric features, such as perimeter, area and centroid while identifying the minimum external geometry. However, the existing algorithms are incapable of achieving this aim. In this study, the method based on the combination of vertex chain code and discrete Green has been adopted, facilitating the rapid extraction of the minimum external geometry of the target image [29].

## 3. Results and Discussion

### 3.1. Extraction of the Contour of Wood Powder Particles

**Figure 2:**
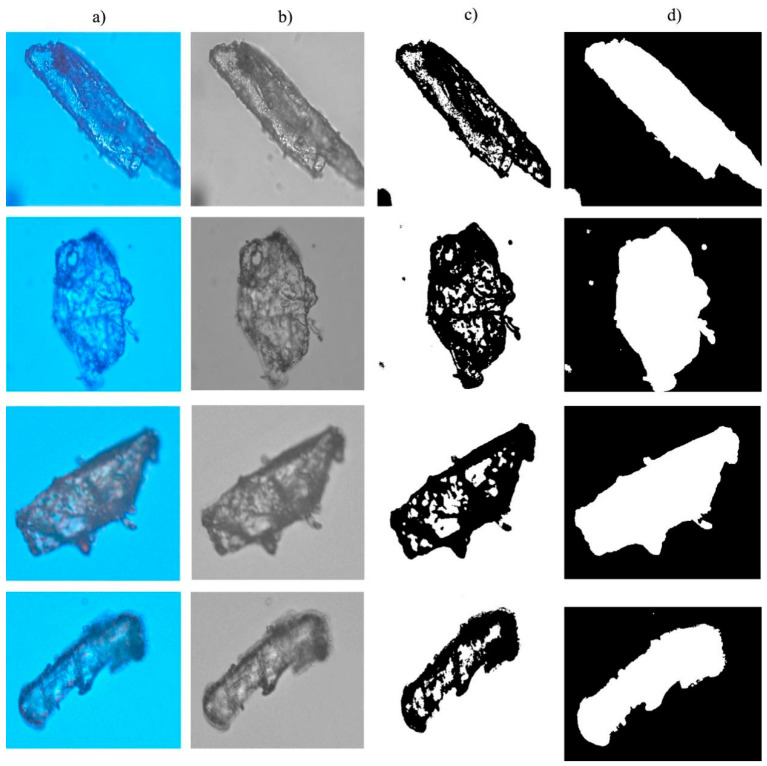
Wood powder particles filling holes: (**a**) Wood flour; (**b**) Grayscale; (**c**) Binary; (**d**) Hole filling.

**Figure 3:**
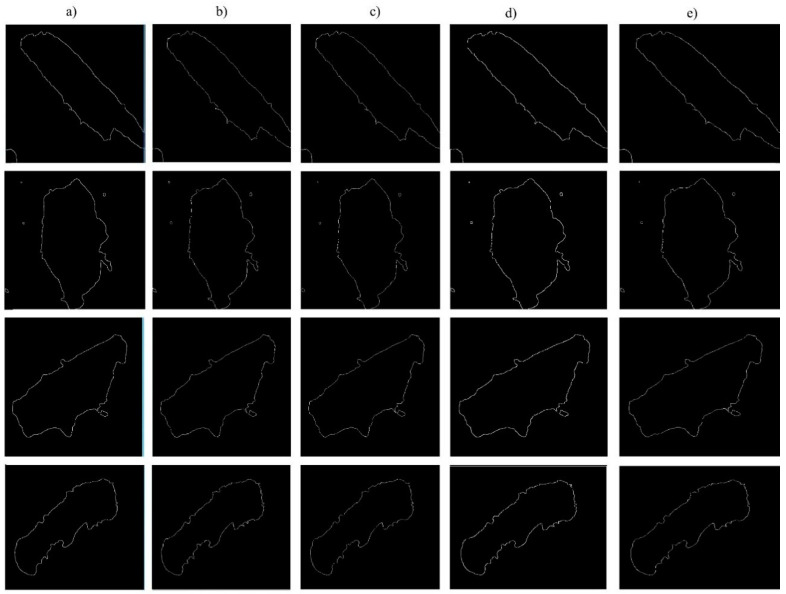
Extraction of the contour of wood powder particles: (**a**) Canny operator; (**b**) Log operator; (**c**) Prewitt operator; (**d**) Roberts operator; (**e**) Sobel operator.

In this study, the commonly used method of contour extraction is adopted to extract the contour of wood powder images. The experimental procedures are illustrated in Figure 2 and Figure 3. The magnification of wood flour image applicable to the experiment amounts to 20 times. Through the experiment, the extraction outline is identified wood powder. Moreover, the unevenly colored microscopic images of wood powder particles could cause holes to experience binarization of gray images and falling phenomenon. In addition, the hole will lead to delayed extraction of the contour of wood powder particles, whereas the contour is not the sole contour due to the discrete issue, which will further lead to the follow-up results of inaccuracy of diameter computation. Therefore, during the experiment, the images are filled subsequent to binarization of wood powder particles with holes, as illustrated in Figure 2. Once the hole filling method is adopted to fill the binary image with holes, the contour of wood powder particles becomes clearer. During the filling of holes, the eight connected regions are analyzed around pixels (x, y). The specific procedures are specified as follows:

First, replicating the original image into the cache before scanning the whole image with a 3 × 3 block centered on the pixel point (x, y) to be filled, and recording the number of points with a gray value of 255 among the 9 points covered by this block with a counter.

Second, identifying the value of counter N. If N ≥ 5, namely, at least 5 of the 9 points belong to the foreground, then the gray value of pixel point (x, y) is assigned to 255, namely, pixel point (x, y) is regarded to belong to the foreground, otherwise it is considered to belong to the background.

Third, replicating the cached image to the original image, and carrying out the processing of mathematical morphology based on the original image.

After hole-filling to remove the impurities around the wood powder particles or the burr of these particles, a series of morphological operations are conducted on their images, such as corrosion and expansion. The decaying candle plays the role of eliminating the target, which is less than the structural element, whereas the inflation plays the role of expanding the pixel, realizing the region expansion and removing the dark details of the images as well while enhancing the edge of the bright region. Subsequently, the operation is turned on to smooth the image and remove bulges and narrow parts. Closed operation can facilitate the connection of gaps and in particular, the narrow ones, in addition to the filling of small holes. The results of the aforementioned operation could vary, depending on which structural element is selected.

Judging from the experiment of wood flour contour extraction, the Roberts operator is able to locate the wood flour contour accurately, but the noise can impose significant impact on the Roberts operator. The Prewitt operator and the Sobel operator are able to extract more than two pixels of wood flour contour, and the two operators feature optimal detection effect for wood flour image with gradual gray gradient and less noise. The accuracy of detecting Log filter’s wood flour contour is inversely proportional to the noise. The better the noise is removed, the lower the wood flour contour accuracy is. The Canny operator is not sensitive to noise and features an optimal effect for weak edge extraction.

Based on the varying quality of images of wood flour particles, different algorithms have been selected for contour detection in the experiment, thus increasing the quality of the contour images of wood flour particles.

### 3.2. Shape Context Matching

**Figure 4:**
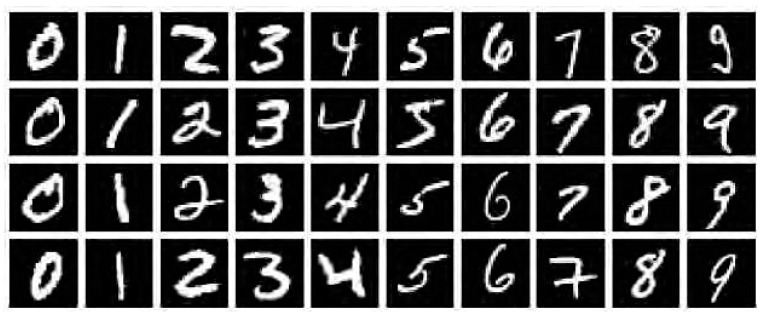
Example of MNIST handwritten dataset.

During the validation experiments in this study, the shape context of MNIST handwritten digital database is adopted for the control experiment in the first place, and the MNIST dataset developed by Yann Lecun is also used. Based on the American National Standards Institute (ANSI), in-depth study is carried out on the handwritten digital data sets. Specifically, the MNIST handwritten grayscale involves 70,000 different people, in each of their pictures, there is a written number ranging between 0 and 9. There are 70,000 images in total, 60,000 for algorithm training and 10,000 for algorithm testing, whereas the image data is a grayscale image of 28 × 8 pixels in essence. An example of a commonly used handwritten number in MNIST is illustrated in Figure 4.

**Figure 5:**
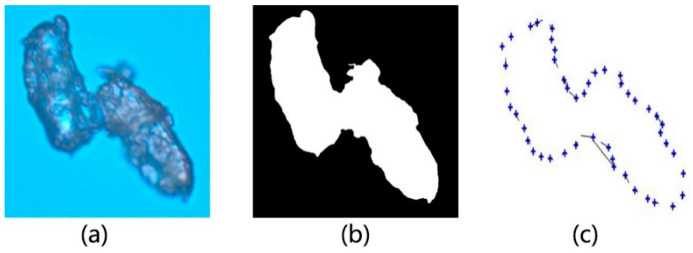
Extraction of Feature points: (**a**) Original image; (**b**) Binary image; (**c**) Feature point.

In general, the shape is represented as a set of points sampled from the shape contour. Typically, 100 points are uniformly sampled, whereas pixel positions are sampled with a boundary detector. Neither is there anything special about these points, nor there is the need to mark or identify the extreme points of curvature, etc. Figure 5 has illustrated the effect picture of feature points extracted through manual writing subsequent to the edge detection and contour tracking. Judging from the experimental analysis, these feature points can still accurately express the contour of the target object after the extraction of feature points.

**Figure 6:**
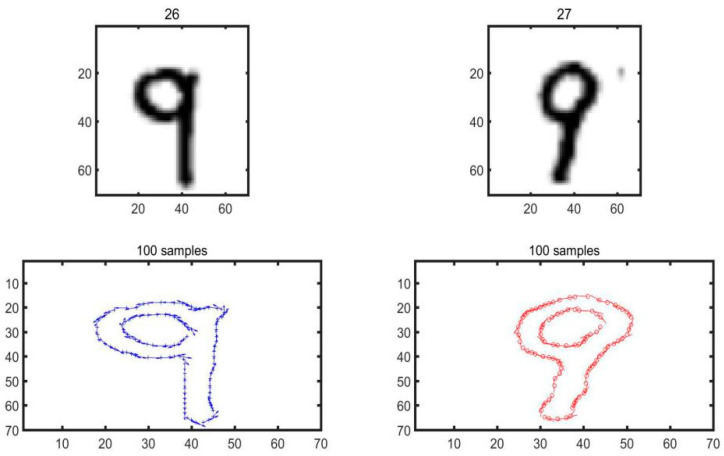
Feature points of the handwritten number ‘9’.

In this study, the feasibility of the algorithm is verified first through the examination of the pictures in MNIST dataset, and two hand-written numbers are selected for the calculation and matching operation through the experiment. Figure 6 has illustrated the outcomes of matching two hand-written numbers ‘9’ and the experimental results of extracting 100 sampling points.

**Figure 7:**
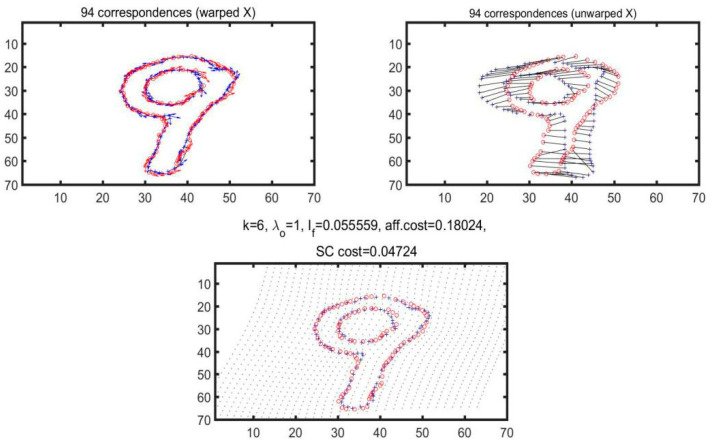
Matching results of handwritten numbers ‘9’.

As illustrated in Figure 6, 100 sampling points are selected to match the number ‘9’ and the matching cost is calculated accordingly, as shown in Figure 7.

During the experiment, the number ‘9’ is reiterated for 6 times, respectively, as specified in Table 1. In addition, such parameters are, respectively, specified, including the number of iterations, corresponding number of points, bending energy minimization, affine transformation and matching cost of shape context.

Judging from Table 1, changes have taken place in bending energy, AFF cost and SC cost during each iteration. However, since the fifth iteration, AFF cost and bending energy did not experience any changes, whereas SC cost still changed. Therefore, based on our analysis, the number of iterations is not the more the better. Instead, it could lead to varying results according to the different matched objects.

**Figure 8:**
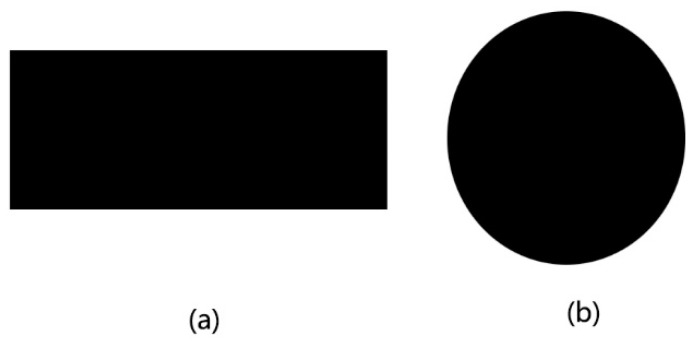
Geometry: (**a**) Square; (**b**) Circular.

Based on the observation of wood powder particles through the experiments, it is found that the microscopic images of wood powder particles most commonly approximate rectangular and circular shapes. Therefore, to facilitate the determination of the geometric shapes of wood powder particles, it is necessary to select the rectangular and circular shapes of wood powder particles for the matching objects, and samples of rectangle and circle objects are illustrated in Figure 8. The analytical results of the matching experiment on wood powder particles with circular and rectangular approximate shapes are specified in Table 2.

Table 2 has shown that varying wood powder particles that match the price stability need to experience different iterations. For some particles, there is the need for repeated iteration to achieve price stability. Moreover, given that the wood powder particles do not abide by the rules of geometry, there is small possibility of matching with rectangular and circular points. As the matching cost elevates, it stands for the mismatch between two shapes. On the contrary, if the matching is less costly, then the shapes are more similar to each other.

### 3.3. Calculation of the Minimum External Geometry

**Figure 9:**
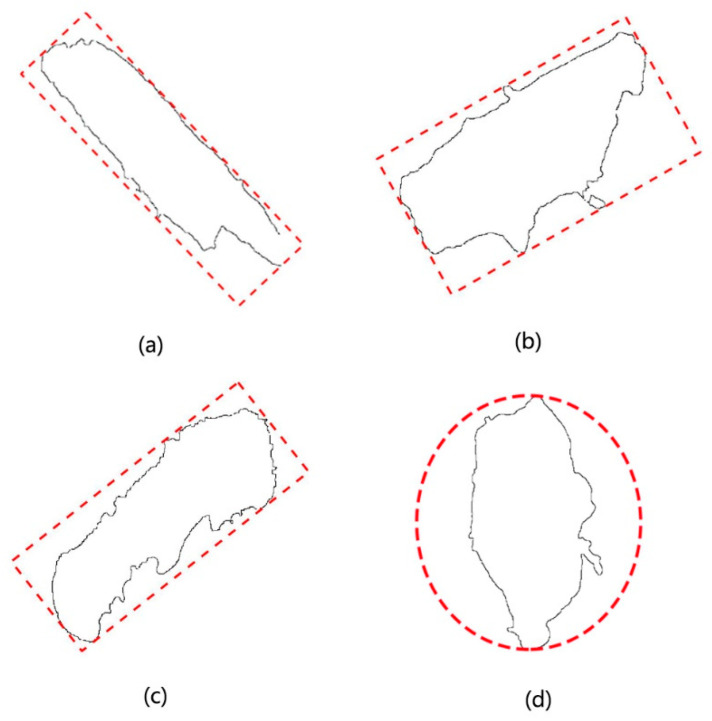
External geometry of Four sorts of wood powder particles: (**a**) Circumscribed rectangle 1; (**b**) Circumscribed rectangle 2; (**c**) Circumscribed rectangle 3; (**d**) Circumscribed circle 4.

Judging from the experimental observation, it is found that the outer contour of most of the wood powder particles are correlated with the geometric figures, whereas the rectangular and circular shapes are the majority. Based on the data specified in Table 2, the approximate result can be obtained with respect to the shape of wood powder particles and rectangular or circular shape. Through the external shape algorithm of the figure, the external geometric figure of wood powder particles is illustrated, as shown in Figure 9.

### 3.4. Calculation of the Diameter of Wood Powders

Mesh is a unit of measuring the diameter. The larger the mesh is, the smaller the diameter is. Numerous standards are available for the conversion between diameter and mesh, such as British standard, Taylor standard and international standard.

Mesh number × aperture (micron number) = 15,000(5)

The software of shooting microscopic images features the function of diameter measurement. During the experiment, the measured values of the software are analyzed and compared with the measured values derived from the method of this study. The analytical results and errors are specified in Table 3 and Table 4.

Set the relative error of diameter as

(6) $$\begin{array}{c} \eta \\ =\left|\frac{\mathrm{Software}\;\mathrm{measurement}-\mathrm{The}\;\mathrm{method}\;\mathrm{adopted}\;\mathrm{in}\;\mathrm{this}\;\mathrm{paper}}{\mathrm{Software}\;\mathrm{measurement}}\right| \end{array}$$

Set the relative error of mesh number as

(7) $$\begin{array}{c} \phi \\ =\left|\mathrm{Software}\;\mathrm{measurement}-\mathrm{The}\;\mathrm{method}\;\mathrm{adopted}\;\mathrm{in}\;\mathrm{this}\;\mathrm{paper}\right| \end{array}$$

**Figure 10:**
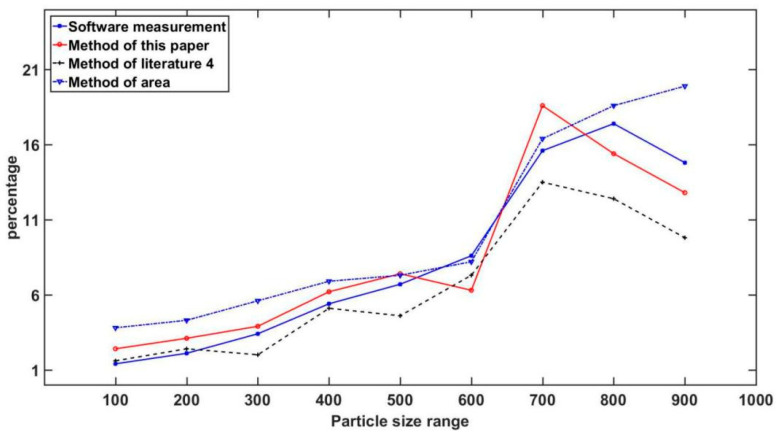
Comparison of wood powder particles.

Subsequent to the comparison of the software measurement method, the image processing method [4], the area method and the method adopted in this paper, the results of the wood powder diameter can be obtained through varying methods, as illustrated in Figure 10. Judging from Figure 10, the diameter distributions calculated by the four methods vary from each other. In terms of the analysis of diameter, different methods of diameter detection and varying formulas of diameter calculation will lead to discrepant results of diameter detection. The more complex the shape of particles is, the more evident the discrepancy in the experimental results will be. The four methods illustrated in the figure have their respective focus. The area method is more applicable to the particles with a spherical shape. However, the majority of the wood powder particles are rectangular, leading to the major discrepancy of the particle distribution between the two methods. Therefore, the method adopted in this paper proves to have better applicability while measuring the number of industrial wood powder mesh. This method is not only applicable to wood powder particles, but also has good generality while measuring and assessing the particles during engineering application, production measurement, scientific research and other practical links of production.

## 4. Conclusions

The method of computing the size of wood flour particles based on geometry fitting put forward in this paper can meet the needs of industrialization, intelligence and automation of the wood flour processing industry. During the experiment, to cope with the issue that the edge of the microscopic image of wood powder is not obvious, a variety of operators are adopted for edge detection to extract the edge, whereas the extraction effects are compared and analyzed accordingly. Given that the holes are prone to emerge during the preparation of wood flour, the diffuse water method is selected during the experiment to fill the binary holes of wood flour, thus resolving the issue of multiple contour and discontinuous contour of wood flour particles. In addition, the shape context is adopted to analyze the shape of wood flour particles, whereas the approximate fitting is carried out for circular and rectangular shapes. Last but not least, the size of wood flour particles can be obtained through the calculation of the side length or diameter of geometric figure. In this study, comparative analysis is conducted on the four methods of calculating the size of wood flour particles. The research findings have shown that this method has good applicability for calculation of the size of wood flour particles, and the method is expected to provide reference for the studies on particle objects in other fields.

## Figures and Tables

**Table 1:** Comparison of the variable value of iteration process.

Number of Iterations	Matching Points	Bending Energy Minimization	AFF Cost	SC Cost
1	81	3.5742 × 10^−6^	0.35099	0.18202
2	91	0.059132	0.20570	0.10958
3	95	0.080640	0.17639	0.052254
4	95	0.078216	0.14836	0.046414
5	95	0.077054	0.15714	0.045859
6	95	0.077054	0.15714	0.046517

**Table 2:** Cost of matching wood powder particles.

	Number of Iterations	Circular				Square			
		Sampling Points	Bending Energy	AFF Cost	SC Cost	Sampling Points	Bending Energy	AFF Cost	SC Cost
Wood powder granules 1	1	37	0.000001	0.77075	0.44625	37	0.000001	0.79356	0.47729
	2	37	0.05495	1.16680	0.23514	37	0.06717	0.86060	0.20302
	3	40	0.03920	1.19980	0.14930	37	0.05572	0.97890	0.17561
	4	42	0.04117	1.18900	0.13052	37	0.03451	0.98428	0.16544
	5	42	0.04378	1.19460	0.12575	38	0.03206	0.98909	0.15200
	6	42	0.04378	1.19460	0.12436	37	0.02592	1.00980	0.15472
Wood powder granules 2	1	37	0.000005	0.45920	0.24640	37	0.000001	0.19076	0.20934
	2	41	0.02717	0.62606	0.16391	39	0.06283	0.35646	0.18307
	3	41	0.02541	0.63584	0.12281	38	0.06929	0.39173	0.15962
	4	40	0.02811	0.63945	0.12104	39	0.07017	0.37001	0.15637
	5	40	0.02804	0.63937	0.11968	38	0.06764	0.38727	0.15848
	6	40	0.02804	0.63937	0.11968	38	0.06850	0.37807	0.16507
Wood powder granules 3	1	37	0.000009	0.56011	0.31992	37	0.0000003	0.55287	0.29962
	2	39	0.04059	0.80981	0.20446	37	0.04057	0.62816	0.21282
	3	45	0.03793	0.84842	0.13430	38	0.05868	0.67458	0.16807
	4	44	0.03775	0.86198	0.12376	39	0.06126	0.61969	0.16481
	5	44	0.03781	0.87760	0.12407	38	0.05728	0.63549	0.15354
	6	44	0.03885	0.89783	0.12386	41	0.06615	0.64970	0.15167
Wood powder granules 4	1	37	0.000003	0.68498	0.41259	37	0.000001	0.64042	0.43226
	2	37	0.07130	0.96566	0.23726	37	0.09679	0.85036	0.29749
	3	37	0.04679	0.94328	0.15079	37	0.08965	0.93991	0.19739
	4	37	0.05537	0.96406	0.14886	37	0.07088	0.98707	0.18840
	5	38	0.05852	1.00540	0.14514	37	0.06786	1.01160	0.17912
	6	37	0.05820	1.03180	0.14626	37	0.06992	1.01380	0.18013

**Table 3:** Values and errors η of the measurement of diameter.

Number of Wood Powder	Software Measurement Value/μm	Measured Value in this Paper/μm	Error
Wood powder particles 1	172.64	168.62	0.02
Wood powder particles 2	127.45	131.64	0.03
Wood powder particles 3	43.36	44.06	0.02
Wood powder particles 4	67.53	66.81	0.01

**Table 4:** Mesh value and error ϕ.

Number of Wood Powder	Calculated Value by Software	Calculated Value in this Paper	Error
Wood powder particles 1	86.89	88.96	2.07
Wood powder particles 2	117.69	113.95	3.75
Wood powder particles 3	345.94	340.44	5.50
Wood powder particles 4	222.12	224.52	2.39

## Data Availability

The study did not report any data.
